# A CT Dataset with RECIST Measurements and Comprehensive Segmentation Masks for Tumors and Lymph Nodes

**DOI:** 10.1038/s41597-026-06597-6

**Published:** 2026-01-20

**Authors:** Roberto Rojas-Pizarro, Constanza Vásquez-Venegas, Gonzalo Pereira, María F. Eyssautier, Felipe Bravo-Bahamóndez, Nicolás Sanhueza, Paulina Gallardo-Badilla, Francisca Caro-Flores, Camila Ormeño-Candia, Felipe Santander, Nicolás Pérez, María M. Molina, Gonzalo Rojas, Steffen Härtel, Guillermo Cabrera-Vives

**Affiliations:** 1 Laboratory for Scientific Image Analysis SCIAN-Lab, Interdisciplinary Nucleus for Biology and Genetics, Institute of Biomedical Sciences ICBM, Faculty of Medicine, University of Chile, Av. Independencia 1027, Santiago, 8380453 Chile; 2 Department of Medical Technology, Faculty of Medicine, University of Chile, Av. Independencia 1027, Santiago, 8380453 Chile; 3 Department of Computer Science, Faculty of Engineering, Universidad de Concepción, Edmundo Larenas 219, Concepción, 4030000 Chile; 4 Radiology Department, Clinical Hospital University of Chile, University of Chile, Dr. Carlos Lorca Tobar 999, Santiago, 8380420 Chile; 5 Centro de Informática Médica y Telemedicina CIMT, Institute of Biomedical Sciences ICBM, Faculty of Medicine, University of Chile, Av. Independencia 1027, Santiago, 8380453 Chile; 6 Biomedical Neuroscience Institute BNI, Faculty of Medicine, University of Chile, Av. Independencia 1027, Santiago, 8380453 Chile; 7 National Center for Health Information Systems CENS, Av. Independencia 1027, Santiago, 8380453 Chile; 8 Centro de Modelamiento Matemático, Universidad de Chile, Santiago, Beauchef 851, Casilla 170-3, Santiago, Chile; 9 Center for Data and Artificial Intelligence, Universidad de Concepción, Concepción, Chile

**Keywords:** Cancer imaging, Machine learning, Data publication and archiving

## Abstract

The Response Evaluation Criteria in Solid Tumors (RECIST 1.1) protocol is the gold standard for assessing treatment response in oncological clinical trials and routine practice. It requires radiologists to review and select appropriate target lesions and perform precise diameter measurements, making the process labor-intensive and variable. Artificial Intelligence (AI) holds great promise for automating this workflow, but progress is hindered by the lack of public datasets with comprehensive lesion annotations and RECIST-compliant measurements. We address this gap by presenting a dataset of 1,246 manually segmented lesions from 58 CT scans of 22 cancer patients treated at the Clinical Hospital of the University of Chile (HCUCH). All cases were evaluated under RECIST 1.1, with diameter measurements reported for 82 target lesions. This resource supports diverse applications, including validating automated RECIST tools, applying radiomics to study metastatic heterogeneity, benchmarking segmentation algorithms, and advancing foundation models in medical imaging. By including data from a Latin American institution, this dataset also promotes global representation in the development of generalizable medical AI tools.

## Background & Summary

Cancer remains one of the leading causes of morbidity and mortality worldwide, posing a significant public health challenge. As reported by the Global Cancer Observatory, nearly 20 million new cases and 9.7 million deaths occurred in 2022, with lung, breast, and colorectal cancers being the most prevalent^1^. While progress in early detection, treatment, and prevention has led to improved outcomes, the strain on healthcare systems is expected to escalate significantly in the coming decades. The global cancer burden continues to grow, driven by factors such as demographic growth, aging, and lifestyle changes, including increased tobacco use, excessive alcohol consumption, unhealthy diets, physical inactivity, and exposure to carcinogenic substances in food and chemical products^2^. Optimizing the clinical workflow in oncologic care is crucial to developing effective and efficient interventions that can manage the rising demand, ensuring sustained access to high-quality and timely healthcare.

Assessment of treatment response is a critical component of the clinical workflow in cancer management. After the initiation of therapy, patients are regularly monitored by healthcare providers to assess whether the cancer is regressing or progressing. In oncological trials and clinical practice, the Response Evaluation Criteria in Solid Tumors (RECIST 1.1) protocol is widely used to quantify tumor burden and track its changes over time, typically using Computed Tomography (CT) imaging. Under this protocol, radiologists identify a small set of representative target lesions and measure the longest diameter of each lesion along the acquisition plane. The tumor burden is calculated as the sum of the longest diameters of the selected target lesions. Throughout the treatment process, these target lesions, and potentially new lesions, are assessed in each follow-up examination. Treatment response is determined by comparing the current tumor burden against the baseline measurement, defined as either the initial assessment or the smallest tumor burden recorded since treatment initiation.

The adoption of the RECIST 1.1 protocol has marked significant progress in standardizing treatment response assessment. However, several challenges continue to impact its effectiveness and efficiency. First, the implementation of RECIST 1.1 suffers from limited reproducibility due to the subjective selection of target lesions and reliance on manual measurements of the longest diameter^3,4^. Second, the estimation of tumor burden is prone to be inaccurate, as it is based on unidimensional measurements rather than volumetric ones^5^. This limitation can lead to underestimation or overestimation of tumor burden, potentially compromising the accuracy of treatment assessments and leading to suboptimal therapeutic decisions for the patient. Lastly, the manual nature of the process is time-consuming, requiring an average of 11 minutes and 30 seconds per assessment^6^, significantly constraining the number of patients a specialist can manage.

Several of these limitations can be mitigated through the use of algorithms that automate the segmentation and measurement of cancer lesions. Because RECIST 1.1 relies on human-driven target-lesion selection, it carries an intrinsic methodological error in estimating total tumor burden, which depends on both the number of lesions and their spatial distribution^7^. Despite this subjectivity, automated segmentation remains valuable, as it can substantially reduce measurement variability and yield more consistent estimations of tumor burden^8^. In this context, Artificial Intelligence (AI) has shown exceptional performance in medical image segmentation^9,10^. However, a critical challenge in training and validating AI models is the collection of expert-annotated datasets, which is particularly labor-intensive for segmentation tasks. Among 14 publicly available datasets for cancer lesion segmentation in CT images (see Supplementary File: *open_datasets_with_tumor_seg_annotations.xlsx*), none provide comprehensive annotations of all observed lesions, including primary tumors, metastases, and lymph nodes. Furthermore, only one dataset^11^ includes lesion measurements consistent with the RECIST 1.1 protocol. Motivated by these gaps, we introduce a dataset comprising 1,246 manually segmented lesions from 58 CT scans of 22 cancer patients treated at the Clinical Hospital of the University of Chile (HCUCH). Each patient was evaluated according to the RECIST 1.1 protocol, and the dataset includes 82 diameter measurements of target lesions as documented in clinical reports. To our knowledge, this is the first dataset to offer instance segmentation masks of all measurable lesions in CT images, spanning primary tumors, metastases, and lymph nodes, alongside corresponding RECIST 1.1 diameter measurements. This dataset provides valuable resources for researchers and industry, supporting applications such as: (i) validating automated RECIST 1.1 workflows, (ii) leveraging radiomics for lesion characterization, (iii) investigating the relationship between volumetric and bidimensional measures, (iv) benchmarking AI models for lesion segmentation, and (v) contributing to the development of foundation models for medical image segmentation, such as MedSAM^12^.

## Methods

### Data collection

**Fig. 1:**
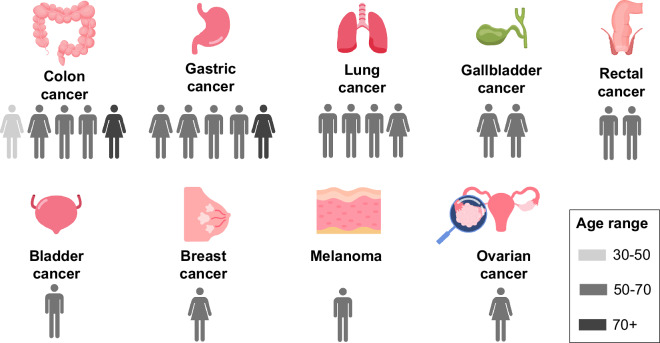
Number of patients, sex, and age in the dataset for different cancer types.

This research and the publication of anonymized, retrospectively collected CT scans were approved by the Scientific Ethics Committee of the HCUCH, acting as the Institutional Review Board (IRB) (Certificate No. 45/23; June 28, 2023). The IRB granted a waiver of informed consent on the basis that all individually identifiable health information had been appropriately removed. Patients were over 18 years of age, had a confirmed cancer diagnosis, and were evaluated according to the RECIST 1.1 protocol. Patients with cancers not assessed by the RECIST protocol were excluded. Only patients with CT scans of the thorax and abdomen/pelvis were included in the analysis. Ethnicity was not included in the dataset, as this information is not captured in the institutional medical records. Following this initial selection, the scans were reviewed by radiologists, and patients were excluded if the images did not contain solid tumors or if the identified lesions were smaller than 10 mm in their largest dimension. As a result, the dataset comprises 58 CT images acquired between 2017 and 2023 from 22 patients diagnosed with various cancer types (Fig. 1). Patient ages range from 37 and 75 years, with a median age of 64. The dataset is balanced by sex, with 11 female and 11 male patients. Most patients do not have health insurance coverage: 16 are uninsured, 5 are covered by the public health system, and 1 by private insurance.

### Data anonymization

All patient-sensitive data were de-identified on our local server immediately after leaving the HCUCH infrastructure. DICOM instances were transmitted from the HCUCH Picture Archiving and Communication System (PACS) in the radiology department, which stores and manages medical images, to a MirthConnect endpoint hosted in our local environment. A curation service then processed the data by removing sensitive metadata such as the Patient’s Address and Birth Date. Meanwhile, the Patient’s Name and Patient ID were pseudonymized, i.e. their original values were replaced with unique integer identifiers to maintain patient differentiation. The original Patient ID was encrypted using the Fernet specification^13^, which employs the AES-128 symmetric encryption algorithm. The encrypted value was then mapped to a unique integer identifier, which serves as the final pseudonymized Patient ID and Name. The mapping between encrypted and integer values was stored in a PostgreSQL table, allowing re-identification only in cases of clinically relevant findings. Finally, all processed DICOM instances were managed by a local Orthanc server.

### CT acquisition and reconstruction

All CT scans were acquired at HCUCH using a standardized protocol on a single clinical scanner (Siemens SOMATON Definition Edge CT, Siemens Healthineers, Knoxville, USA), with patients positioned supine on the scanning table. Each CT scan corresponds to a distinct imaging study conducted for a patient. In total, 48 studies were included across the 22 patient cohort. Each patient contributed between one and four studies, with the first serving as the baseline and subsequent ones as follow-ups. For each study, multiple series were reconstructed slice by slice in the axial plane as volumetric CT images, each covering a distinct anatomical region (e.g. thoracic, abdomen and pelvis). For this dataset, only thoracic and abdominal series were selected, resulting in 22 thoracic and 36 abdominal CT images containing measurable tumor lesions. The acquisition and reconstruction parameters for each series type are detailed in Table 1.

**Table 1:** CT acquisition parameters for each anatomical region (thorax and abdomen).

Parameter	Thorax	Abdomen
Contrast Agent	No contrast	125 ml intravenous, portal-venous phase
CareDose	Connected	Connected
Tube Voltage (kV)	120	100
Reference Tube Current-Time Product (mAs)	66	180
Rotation Time (s)	0.5	0.5
Pitch Factor	1.2	1
Collimation (mm)	128 × 0.6	128 × 0.6
Scan Range	Lung apex to Suprarenal glands	Diaphragmatic domes to pubic symphysis
Slice Thickness (mm)	1.5	5.0
Reconstruction Kernel	I70f	I30f

**Fig. 2:**
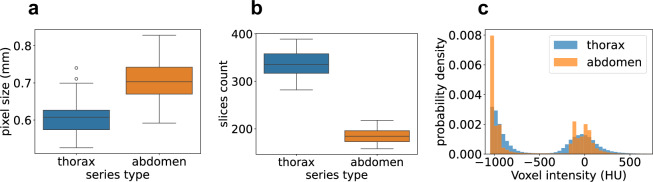
Properties of CT images. (**a**) Pixel sizes by series type. Slice spacing is 1.0 mm for thoracic and 2.5 mm for abdominal series. (**b**) Slice counts by series type. All slices have a fixed in-plane resolution of 512 × 512 pixels. (**c**) Probability distributions of voxel intensities by series type. Each distribution represents the mean of individual CT scan intensity distributions.

The reconstructed CT images resulted in a slice dimensions of 512 × 512 pixels and a slice count ranging from 158 to 389. The pixel size in the axial plane varies across scans, with a mean value of 0.67 ± 0.07 mm (range: 0.53–0.83 mm). Thoracic CT images have higher resolution than abdominal images and contain nearly twice as many slices (Fig. 2). Specifically, thoracic series have a mean pixel size of 0.61 ± 0.06 mm (range: 0.53–0.74 mm), with slice counts ranging from 282 to 389 (IQR: 317–358), whereas abdominal series have a mean pixel size of 0.7 ± 0.06 mm (range: 0.59–0.83 mm) with a number of slices between 158 and 218 (IQR: 173–196), as shown in Fig. 2a and b. The spacing between slices of the reconstructed CT images of thorax is 1 mm, while for abdomen is 2.5 mm. These values differ from the slice thicknesses used during acquisition (1.5 mm for thorax, 5.0 mm for abdomen) due to overlapping slices in the z-dimension during scanning. Finally, the voxel intensity of CT, expressed in Hounsfield Units (HU), was analyzed. HU are a scale used in CT imaging to measure the attenuation (and hence, density) of tissues based on how much X-ray radiation they absorb. This scale allows for quantitative differentiation of tissues in medical imaging. As it is observed in Fig. 2c, probability distributions of voxel intensities from thorax and abdomen CT images are similar, presenting a bimodal behaviour around values of −1000 HU (air) and 0 HU (water).

### DICOM-to-NIfTI conversion

Conversion from DICOM to NIfTI format was performed based on the *dcm2niix* utility (https://github.com/rordenlab/dcm2niix), resulting in a single compressed NIfTI file for each CT series volume.

### CT Windowing

Windowing is an image processing technique used in radiology to highlight specific anatomical structures for visualization purposes. It involves mapping original pixel values within a predefined range of HU, known as the window, to a grayscale range of 0 to 255. Values below the window are set to the minimum value 0 (black), while values above the window are set to the maximum value 255 (white). Pixel values within the window range are linearly mapped to intermediate grayscale intensities. The window is defined by the interval [L-W/2, L + W/2], where L is the window level (center) and W the window width. Although there is no universal standard, radiologists typically rely on consistent window settings to interpret specific anatomical regions. In this study, Windowing was applied to thoracic series using a lung window (L: −500, W: 1400), and to abdominal series using an abdomen window (L: 40, W: 350). Exactly the same standardized windowing settings were applied: (i) for qualitative visualization, (ii) during data labeling to minimize interobserver variability between radiologists, and (iii) as a preprocessing step for Deep Learning (DL)-based lesion segmentation, as detailed in the Technical Validation section.

### Data labeling

CT images were assessed by two radiologists with over 5 and 10 years of experience, along with a group of 9 radiology residents. Patients were divided into training and test sets, with 14 patients assigned for training and 8 for testing. The test set was manually annotated from scratch by the two expert radiologists, and any discrepancies were resolved through a consensus process to ensure consistency and reliability.

**Fig. 3:**
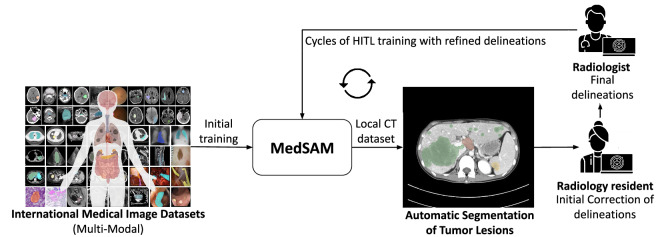
Human-in-the-Loop (HITL) cycles for assisted annotation of CT images. MedSAM foundational model is used as an AI-assistant to generate initial segmentations from bounding box prompts drawn by radiology residents. MedSAM segmentations are corrected by radiology residents and refined by radiologists. The improved masks are used to retrain the MedSAM model before starting a new HITL cycle.

For the training data, a Human-in-the-Loop strategy^14^ was used to streamline the annotation process (Fig. 3). This AI-assisted method leveraged MedSAM^12^, a DL foundational model pre-trained on public medical imaging datasets, for initial lesion segmentations. First, a radiology resident placed a 3D bounding box around tumor lesions, prompting MedSAM to generate automated segmentations. These segmentations were then refined through a hierarchical two-step review: radiology residents performed initial corrections, which were subsequently reviewed and finalized by the expert radiologists. Manual delineations were performed using 3D Slicer, where MedSAM was integrated as a plugin for segmentation assistance.

After each batch of images was annotated and corrected, the DL model was retrained to incorporate the refined segmentations, progressively improving its performance in terms of Dice score. With each iteration, the need for manual corrections decreased as the model adapted to the local dataset. The retraining process continued until the model’s performance exceeded the baseline quality expected by expert radiologists, as evaluated using the independently annotated test set. Results are detailed in the Technical Validation section.

Regarding the labeling protocol, each delineated lesion was assigned a label consisting of two comma-separated values: the first indicating the lesion type (*t* for primary tumors, *m* for metastases, and *n* for adenopathies) and the second specifying its location (the affected organ for solid tumors or the anatomical region for lymph nodes).

RECIST measurements of target lesions were also recorded. Each patient’s target lesions were assigned a unique capital letter identifier (A, B, C, …) to enable longitudinal tracking across follow-up studies. The integer value corresponding to each target lesion in the segmentation mask was also documented to maintain correspondence between RECIST measurements and delineated lesions.

### Data properties

**Fig. 4:**
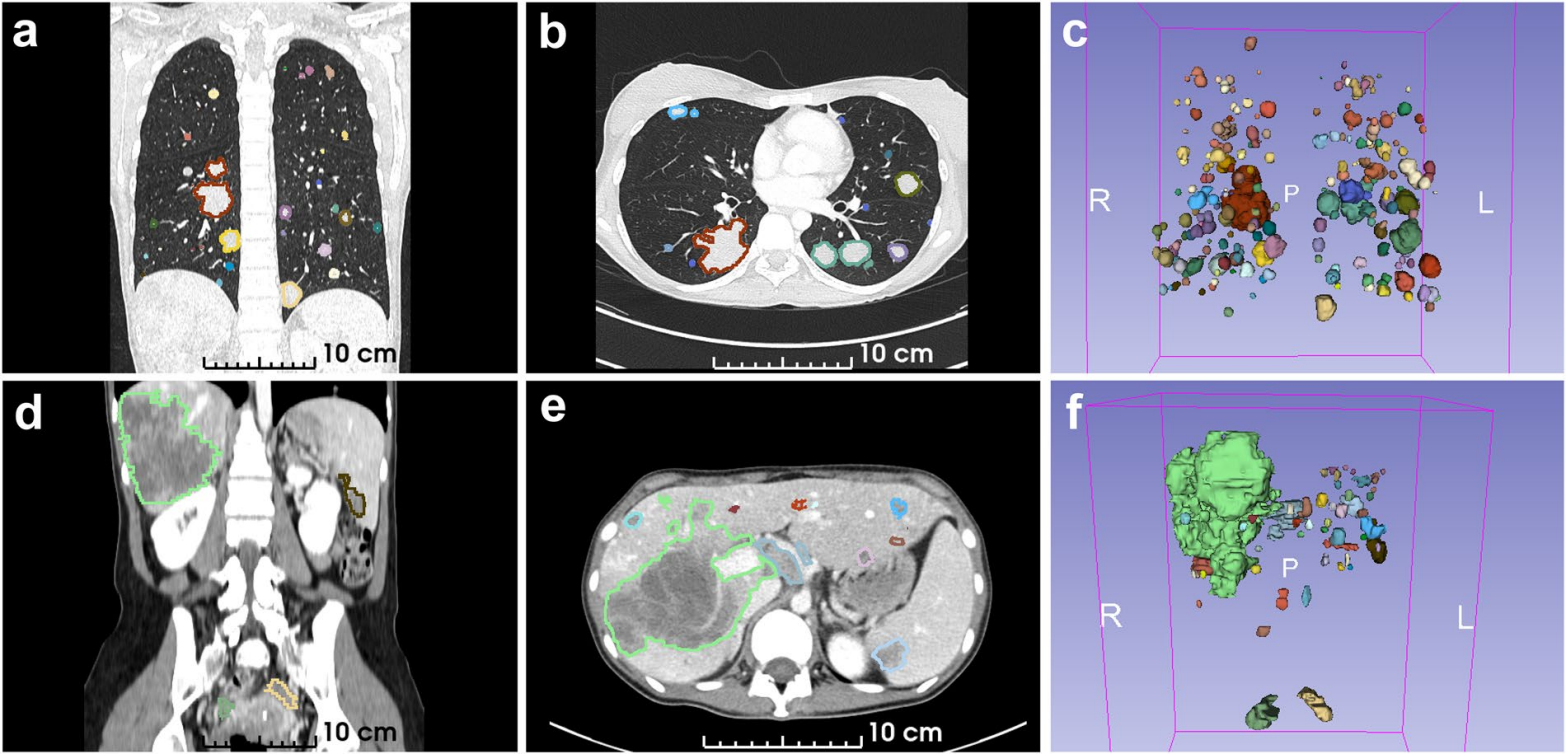
Examples of annotated lesions in CT images. Each row corresponds to a series obtained from the same patient and CT study. (**a**–**c**) Thorax. (**d**–**f**) Abdomen. Each column shows a specific view of the CT image, displaying annotated lesions in color. From left to right: coronal plane, axial plane, and 3 d rendering.

The annotated segmentation masks were preprocessed to obtain the final set of lesion instances. Each mask was normalized so that all foreground voxels within the same label category shared the same voxel value. Lesion instances were then identified as connected components of the same foreground value, using full voxel connectivity (26-connectivity). To suppress annotation artifacts, connected components smaller than 50 voxels were removed. This process resulted in a total of 1,246 delineated lesions, including 1,148 metastases, 93 enlarged lymph nodes (adenopathies), and 5 primary tumors. The majority of tumors were located in the lungs (939 tumors) and liver (192 tumors). Figure 4 presents representative thoracic and abdominal CT images with examples of delineated lesions, while Supplementary Table 1 provides detailed summaries of the annotated lesions per patient and lesion label.

**Fig. 5:**
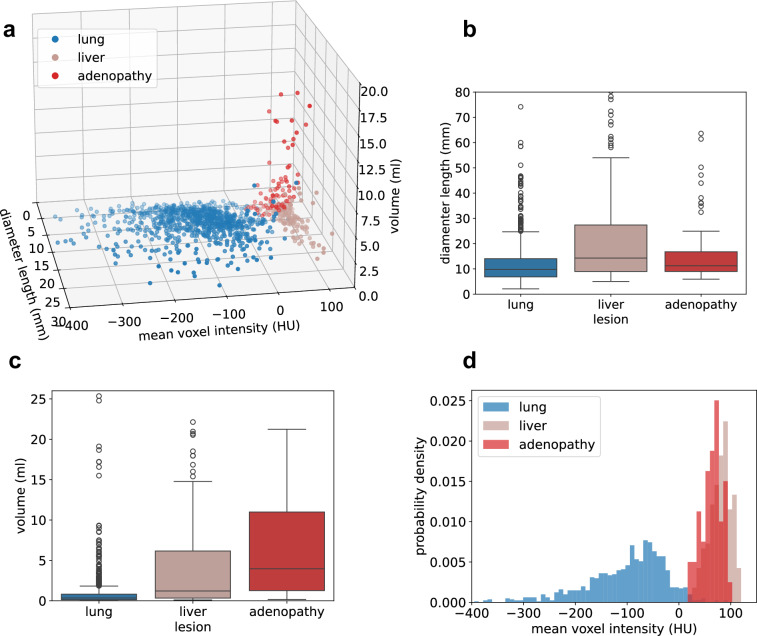
Lesion properties of most represented lesions. (**a**) Scatterplot of lesions in the feature space defined by diameter length, mean voxel intensity and volume. (**b**) Boxplot of lesion diameter lengths, showing major diameters for tumors and minor diameters for adenopathies. (**c**) Boxplot of lesions volumes. (**d**) Probability distributions of lesions mean voxel intensities. Outliers were omitted from panels (**a**–**d**).

The delineated lesions were analyzed based on three key features: volume, diameter length, and mean voxel intensity. For tumors, diameter length was computed as the largest major axis of the minimum-area, oriented bounding box enclosing the lesion in any axial slice. For adenopathies, it was calculated as the minor axis of the slice with the largest major axis. Further analysis was conducted on the most prevalent lesion types: lung tumors, liver tumors, and adenopathies (Fig. 5). Lung tumors were the smallest in terms of volume and largest diameter, with median values of 0.4 ml (IQR: 0.1–0.8 ml) and 9.8 mm (IQR: 6.8–14.0 mm), respectively. Adenopathies tended to be larger in volume than liver tumors, with median values of 4.0 ml (IQR: 1.3–11.0 ml) and 1.2 ml (IQR: 0.3–6.2 ml), respectively. However, in terms of largest diameter, liver tumors were larger than adenopathies, with medians of 14.2 mm (IQR: 9.0–27.3 mm) and 11.2 mm (IQR: 9.0–16.8 mm), respectively.

Regarding voxel intensities, the mean HU computed over the lesions resulted in probability distributions exhibiting shifts in their observed means (−101.5 ± 92.0 HU for lung, 67.8 ± 19.0 HU for adenopathies, and 84.3 ± 20.2 HU for liver). Notably, the considerable difference between the lung distribution and those of adenopathies and liver tumors highlights a potential for differentiation, reinforcing the relevance of radiomics in identifying additional predictive or discriminative features.

The correlation between volume and diameter length was assessed using Spearman’s test, revealing a strong positive monotonic relationship across all lesions (*ρ* = 0.92, p < 1e-300). When analyzed by lesion type, the correlation remained similarly strong: *ρ* = 0.91 for adenopathies (p = 9.9e-36), *ρ* = 0.96 for liver tumors (p = 2.3e-104) and *ρ* = 0.95 for lung tumors (p < 1e-300). This statistical relationship is crucial, as it represents a fundamental prerequisite for using diameter length as a proxy for tumor volume and, consequently, for tumor burden assessment.

**Fig. 6:**
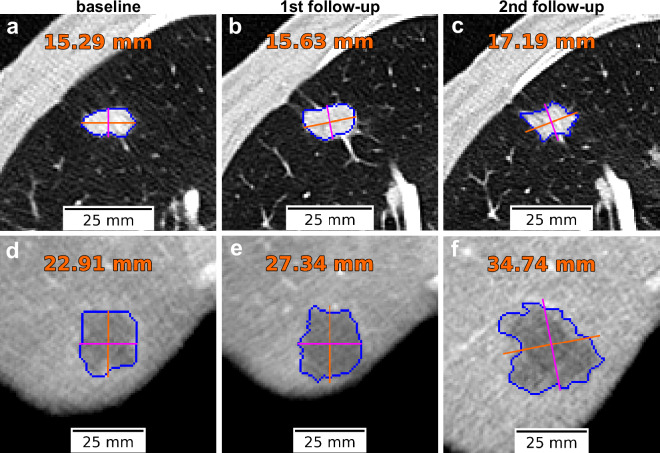
Target lesion evolution over time, with automatically computed diameter lengths. (**a**–**c**) Lung metastasis. (**d**–**f**) Liver metastasis. The left column corresponds to the baseline study, while middle and right columns show subsequent follow-up studies. Major and minor axes are displayed in orange and magenta, respectively. The diameter length in millimeters, corresponding to the major axis in this case, is shown in orange.

A total of 82 target lesions were measured and documented in the RECIST report, comprising 29 liver tumors, 22 lung tumors, and 23 adenopathies. The remaining lesions correspond to tumors located in the ovary, abdominal wall, kidney, and adrenal gland. Figure 6 illustrates the temporal evolution of target lung and liver lesions, displaying the baseline study (left) alongside two subsequent follow-ups (middle and right). For each lesion, the automatically computed largest diameter is shown. These measurements, derived from segmentation masks, can be compared to RECIST-based assessments, offering insights into the potential of automated methods to streamline clinical workflows for tumor response evaluation.

A summary of the dataset composition, including the number of patients, CT studies and series, segmented lesions, and RECIST measurements, is provided in Table 2.

**Table 2:** Summary of patients, imaging data, lesion annotations, and RECIST measurements in the dataset.

Description	# Count
# patients	22
# studies	48
# CT series	58
# annotated lesions	1246
# RECIST measurements	82

## Data Records

The dataset is available on Zenodo (https://zenodo.org/records/17788162) under a Creative Commons Attribution 4.0 International (CC BY 4.0) license^15^. The data is structured as described below.

### Imaging data

The dataset comprises 58 CT series and their corresponding lesion-instance segmentation masks, organized by anatomical region (*abdomen* and *thorax*) and divided into *train* and *test* subfolders. Each of these subfolders contains three subdirectories: *images*, which contains the CT series in compressed NIfTI format (.nii.gz); *masks*, which stores the segmentation masks in the same format; and *labels*, which includes JSON files with the mapping between foreground voxel values and lesion labels for each segmentation mask. Corresponding CT series, mask, and label files are identified by their filenames, which share a common basename that combines the *Patient ID* with the original DICOM *Series Instance UID*.

### Metadata

Patient-level metadata is provided in the file *patients.csv*, which contains diagnostic and demographic information. Series-level metadata is stored in *series.json*, including image acquisition properties and study dates. Additionally, *windows_mapping.json* is included to facilitate intensity normalization via windowing.

### RECIST measurements

RECIST 1.1 measurements are provided in the file *recist_measurements.csv*, reporting the unidimensional size of 82 target lesions annotated in the dataset. In accordance with RECIST 1.1, the recorded measurement corresponds to the major axis for tumors and the minor axis for adenopathies, all assessed in the axial plane. The tracking of target lesions was manually verified on a lesion-by-lesion basis by the most experienced radiologist (GP). None of the 82 target lesions were split, merged (coalesced), or disappeared over time.Lesion appearance across time points can be inspected using the *lesion_label_alias* column in *recist_measurements.csv*. Table 3 summarizes all variables in this file as well as those in the additional metadata files described above.

**Table 3:** Summary of the content of CSV and JSON files included in the dataset.

Filename	Description of headers
patients.csv	Patient ID, Subset, Date of baseline study, Biological sex, Age at the moment of baseline, Clinical diagnosis, Histological diagnosis, Health insurance coverage
series.json	Series ID, Anatomical region, Series Instance UID, Study Instance UID, Date of the study, Patient ID, Slice thickness during acquisition (mm), Voxel size in row dimension (mm), Voxel size in column dimension (mm), Voxel size in slice dimension (mm), Number of slices, Number of rows, Number of columns
windows_mapping.json	Filename of CT series, window name
recist_measurements.csv	Patient ID, Subset, Date of study, Study Instance UID, Series Instance UID, Filename of CT series, Anatomical region, Total number of annotated lesions in the CT series, Foreground value, Alias to identify lesion within the patient, RECIST diameter measurement (mm), Temporal category of the study

A detailed description can be found on Zenodo.

## Technical Validation

### Deep Learning-based semi-automatic lesion segmentation using MedSAM

The first technical validation focused on fine-tuning the MedSAM model to assist in annotating the proposed dataset using the HITL strategy described in Fig. 3. Each HITL cycle produced a batch of manually labeled CT images used to refine MedSAM. The model was updated only if the fine-tuned version outperformed the previous one on the manually annotated HCUCH test set.

#### Preprocessing

CT images were properly normalized using the window settings previously specified in the Methods section. As MedSAM is designed for 2D segmentation, only slices containing annotated lesions were extracted from the training and test CT images. To match MedSAM’s input requirements, image slices were resized to 1024 × 1024 x 3 using third-order spline interpolation, while mask slices were resized to 1024 × 1024 using nearest-neighbor interpolation.

#### Training

Only the MedSAM decoder was fine-tuned, keeping the encoder weights frozen. After each cycle, all available annotated CT images were used to fine-tune MedSAM, starting from its original pre-trained weights. Each training sample consisted of a CT slice paired with the bounding box of a randomly selected 2D connected component from the segmentation mask. To improve robustness, bounding boxes were perturbed by 0–5 pixels at each corner. Leave-One-Patient-Out (LOPO) cross-validation was used, selecting the best model for each validation patient. The following hyperparameters were used for each training run: 50 epochs, batch size equal to 2, initial learning rate of 1e-4 and weight decay of 0.01.

#### Evaluation

All 2D connected components from the annotated CT images in the HCUCH test set were used for evaluation. The optimal bounding boxes enclosing each component served as prompts to generate segmentations from the fine-tuned models. Table 4 summarizes the number of annotated CT images used for training, the total number of extracted lesions, and 2D connected components with at least 10 pixels. The final model from each HITL cycle corresponds to the LOPO cross-validation fold that achieved the highest performance on the test set. The test set includes 20 annotated CT images, 589 lesions, and 5,248 2D connected components. Cycle 0 represents the original pre-trained MedSAM model.

**Table 4:** Segmentation performance obtained by MedSAM after 3 HITL cycles.

Cycle	Total training CT images	Total training Lesions	Total training 2D connected components	Dice score	
				*μ* ± *σ*	Med (5th-95th)
0	—	—	—	0.84 ± 0.13	0.88 (0.58–0.96)
1	13	354	4135	0.88 ± 0.07	0.90 (0.74–0.96)
2	24	438	4947	0.88 ± 0.08	0.89 (0.73–0.96)
3	38	576	7348	0.88 ± 0.08	0.90 (0.74–0.96)

**Fig. 7:**
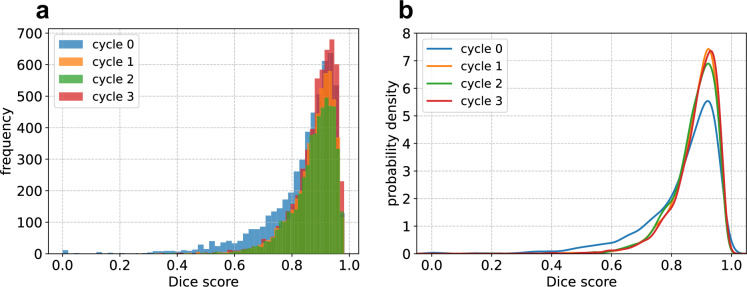
Distribution of Dice scores obtained by MedSAM after each HITL cycle. (**a**) Histograms. (**b**) Probability densities estimated using Gaussian Kernels.

**Fig. 8:**
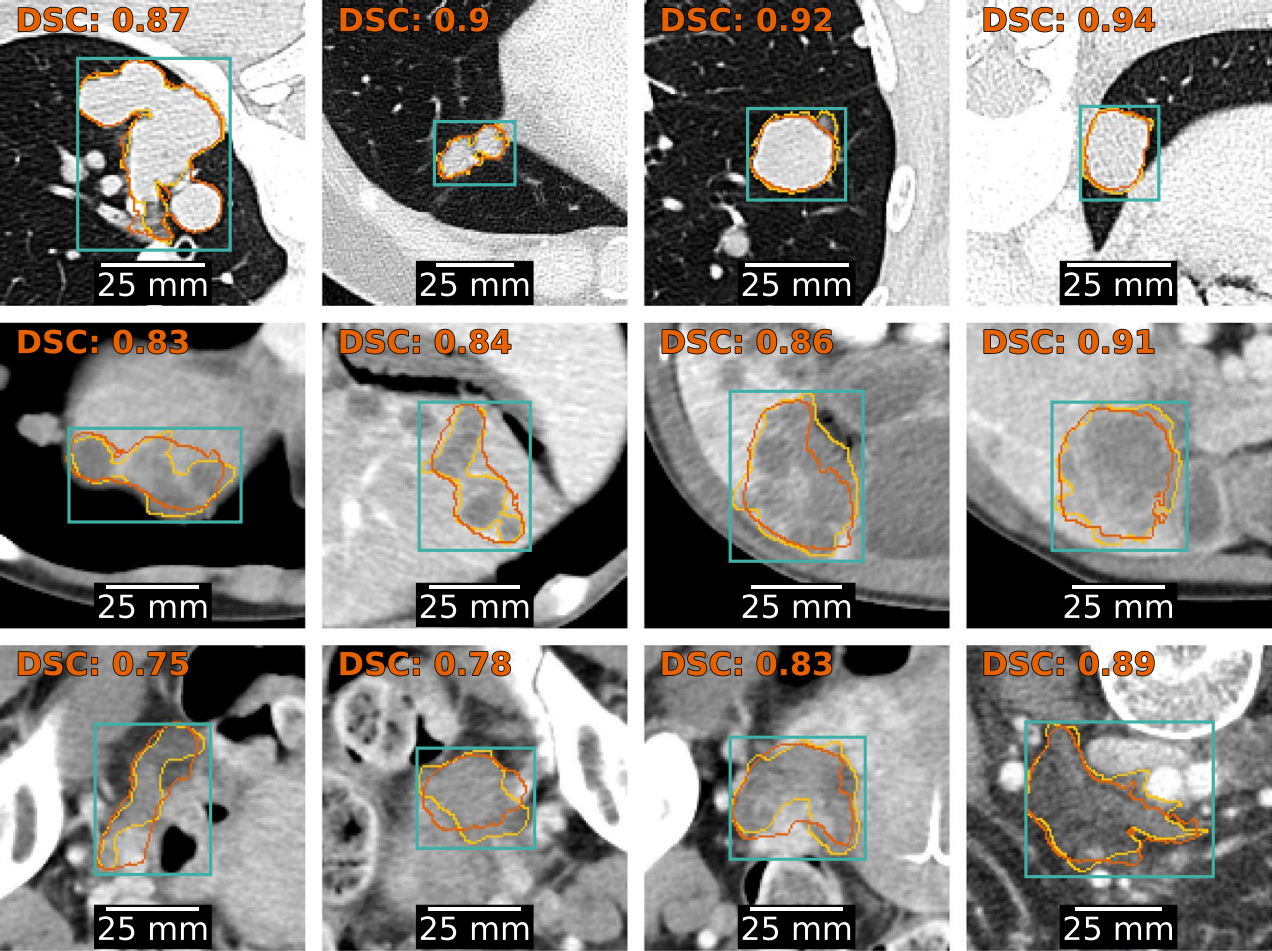
Visual examples of MedSAM segmentations after three HITL cycles. Representative segmentations with Dice scores within the interquartile range for three lesion types. The top row presents lung tumors, the middle row liver tumors, and the bottom row adenopathies. Each image includes the bounding box provided to the model (cyan), the contour of the ground truth delineation (yellow), the contour of the MedSAM segmentation (orange), and the corresponding Dice score (orange, top left corner).

All HITL cycles achieved statistically significant improvement in Dice scores (p < 3.7e-7, Wilcoxon test). This improvement is reflected in a rightward shift of the Dice score distributions across cycles (Fig. 7). The most substantial improvement occurred in the first cycle, with a median Dice score increase of 0.02 (IQR: 0–0.06). After 3 cycles of HITL, MedSAM achieved an overall median improvement of 0.03 (IQR: 0–0.08) compared to its original version. By lesion type, the median Dice scores were (IQR: 0.87–0.94) for lung tumors, 0.88 (IQR: 0.83–0.91) for liver tumors and 0.83 (IQR: 0.74–0.89) for adenopathies. Representative segmentations from the test set at the final training cycle are shown in Fig. 8.

This study demonstrates the potential of the proposed dataset for assisted lesion segmentation using bounding box prompts. The achieved performance aligns with MedSAM’s internal validation for segmenting lung tumors, liver tumors, and lymph nodes^12^. Beyond assisted segmentation, this dataset could be integrated into MedSAM’s original training set to enhance future iterations of the model. It may also contribute to the development of new medical foundational models, extending its impact on AI-driven medical image analysis.

### Deep Learning-based automatic tumor segmentation using nnUNet

The second technical validation involved training and evaluating a DL model for automatic tumor segmentation. The nnUNet framework^16^ was selected for its standardized and automated pipeline for developing U-Net-based models tailored to medical image segmentation. To incorporate prior knowledge and improve generalization, the model was initialized with weights pre-trained on CT data from the Medical Segmentation Decathlon (MSD)^17^, and subsequently fine-tuned on the proposed dataset. Separate models were trained for lung and liver tumor segmentation.

#### Preprocessing

All CT images were preprocessed using standardized windowing as described in the Methods section. For the segmentation masks, only lung and liver tumor segmentations (primary and metastatic) were retained. All other delineated structures, including additional lesions or organs (in the case of MSD datasets), were excluded from the segmentation masks. The resulting masks were then binarized, setting all positive voxels as foreground (tumor).

#### Training

Pre-training was conducted using the MSD-Lung and MSD-Liver datasets, which include segmentation masks for primary lung tumors, and for both primary liver tumors and liver metastases. An overview of these MSD datasets, along with the HCUCH dataset used for fine-tuning, is presented in Table 5.

**Table 5:** Datasets used to fine-tune nnUNet on HCUCH data for automatic segmentation of lung and liver tumors. nnUNet was initially pre-trained on datasets from the Medical Segmentation Decathlon (MSD).

Property	MSD-Lung	HCUCH-Lung	MSD-Liver	HCUCH-Liver
Patients	—	10 (8/2)	—	9 (7/2)
Images	63	21 (16/5)	131	21 (16/5)
Lesions	70	939 (412/527)	857	192 (104/88)
Annotated Volume (ml)	1384.8	976.1 (557.6/418.5)	10213.2	3158.6 (1033.4/2125.2)

All CT images from the MSD were used for pre-training, while the HCUCH dataset was split into training and testing sets, as indicated in parentheses (training/testing). Patients included in the MSD datasets are not reported.

During pre-training, all hyperparameters remained at their default settings: 1000 epochs, an initial learning rate of 0.01, and a weight decay of 3e-5. Patch sizes, batch sizes, and network parameters were automatically determined for a 3D configuration following the nnUNet pipeline. Pre-training utilized the entire MSD dataset to obtain a base model without a training-validation split. For fine-tuning, 5-fold cross-validation was performed on the HCUCH training set, generating a best model for each fold. These models, along with their ensemble, were then evaluated on the HCUCH test set. Segmentation performance was assessed by calculating the Dice score for each CT image. For fine-tuning, the training runs were modified to have 300 training epochs and a smaller initial learning rate equal to 1e-4.

#### Evaluation

**Fig. 9:**
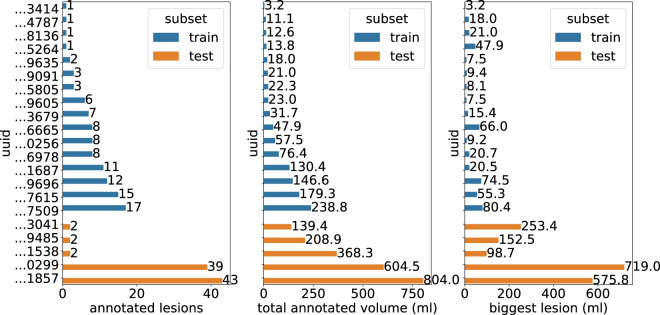
Characterization of CT images used for fine-tuning the nnUNet model for liver tumor segmentation. Each CT image is uniquely identified by its DICOM Universally Unique Identifier (UUID). The last test case (UUID ending in …0299) contains a markedly large tumor with a volume of 719.0 ml, substantially exceeding the tumor volumes observed in the training set.

**Fig. 10:**
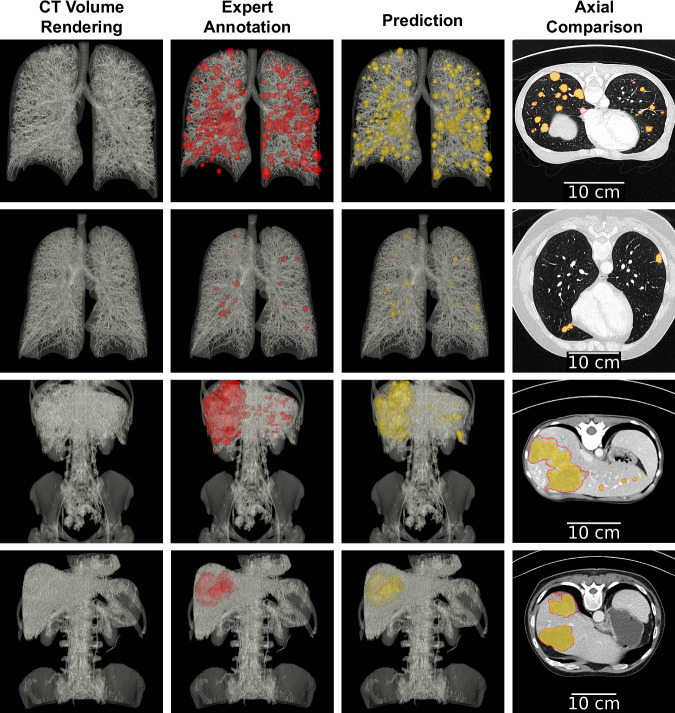
Segmentations produced by the nnUNet fine-tuned on the HCUCH dataset for automatic lung and liver tumor segmentation. From left to right, the first three columns show: (1) 3D rendering of the CT volume from the coronal view, (2) CT overlaid with expert annotations (red), and (3) CT overlaid with nnUNet predictions (yellow). The fourth column presents an axial slice with expert annotation contours and the filled predicted mask. Each row represents a different patient from the test set; the first two correspond to thoracic series and the last two to abdominal series.

Dice scores on the test set are summarized in Table 6. For lung tumor segmentation, all test CT images exhibited substantial improvements following fine-tuning, with Dice score increases ranging from 0.11 to 0.33. In contrast, for liver tumor segmentation, only one test case demonstrated a notable improvement of 0.13, while the remaining four maintained performance comparable to the pre-trained model. This consistency is likely due to the strong baseline performance of the pre-trained model, which already achieved Dice scores above 0.83. Notably, the only liver case with poor baseline performance, which also showed the greatest improvement, involved a very large tumor (719 ml). This tumor was considerably larger than any observed during training, with the largest observed tumor measuring 80.4 ml (Fig. 9), likely contributing to the model’s difficulty in segmenting it accurately after fine-tuning. Representative segmentation outputs obtained by the final fine-tuned models are shown in Fig. 10.

**Table 6:** Dice scores on testing CT images from HCUCH obtained by the ensembles of the fine-tuned nnUNet for lung and liver tumor segmentation.

CT image	tumor	Pre-trained Dice score	Fine-tuned Dice score	Improvement
…1523	lung	0.55	0.78	0.23
…9660	lung	0.52	0.85	0.33
…2721	lung	0.75	0.86	0.11
…5769	lung	0.72	0.86	0.14
…7846	lung	0.76	0.88	0.12
…1857	liver	0.83	0.87	0.04
…3041	liver	0.91	0.92	0.01
…0299	liver	0.27	0.40	0.13
…9485	liver	0.93	0.93	0
…1538	liver	0.91	0.88	−0.03

These results highlight the suitability of the proposed dataset for training DL models in automatic segmentation of lung and liver tumors. Fine-tuning led to notable performance gains in lung tumor segmentation, with Dice scores on the test set ranging from 0.78 to 0.88. For liver tumor segmentation, the model maintained strong performance, achieving Dice scores between 0.87 and 0.93, excluding the previously discussed outlier with an unusually large tumor. These outcomes surpass the Dice scores reported by nnUNet on the MSD datasets (0.74 for MSD-Lung and 0.76 for MSD-Liver), as described by Isensee *et al*.^16^, underscoring the value of the proposed dataset for advancing tumor segmentation models.

## Supplementary information

Supplementary Material

Open datasets with annotations of tumors

## Data Availability

The dataset is available on Zenodo (https://zenodo.org/records/17788162) under a Creative Commons Attribution 4.0 International (CC BY 4.0) license^15^.

## Code availability

All code used for data conversion, windowing normalization and statistical analysis is available at https://github.com/robertorojasp06/recist-dataset. Code for fine-tuning and evaluating nnUNet is available at https://github.com/robertorojasp06/nnUNet. Code for experiments using MedSAM is available at https://github.com/robertorojasp06/MedSAM.

All code for dataset preprocessing and characterization was implemented in Python (3.10) on Ubuntu 22.04.5 LTS. The specific Python packages used are listed in the environment.yml file of the recist-dataset repository available at https://github.com/robertorojasp06/recist-dataset. Experiments involving nnUNet and MedSAM were conducted using the same package versions specified in their respective GitHub repositories. All experiments were run on a machine equipped with an AMD Ryzen 7 5700 G processor and an NVIDIA GeForce RTX 4090 GPU.
