# Supplementary material for: A CT Dataset with RECIST Measurements and Comprehensive Segmentation Masks for Tumors and Lymph Nodes
Source: Sci Data. 2026 Jan 20;13:270. doi: 10.1038/s41597-026-06597-6 (PMC12917031; doi:10.1038/s41597-026-06597-6)
Supplement: Supplementary file 1 — Supplementary Material [file 41597_2026_6597_MOESM1_ESM.pdf]

subset	patient id	diagnosis	lesions count	annotated volume (ml)	annotated lesions
train	1	lung cancer	3	154.1	t,lung (1)<br>m,kidney (1)<br>n,mediastinum (1)
train	2	gastric cancer	3	13.9	m,liver (2)<br>m,lung (1)
train	5	colon cancer	1	3.2	m,liver (1)
train	7	rectal cancer	22	130.4	m,liver (22)
train	8	gastric cancer	47	216.8	m,liver (24)<br>m,lung (12)<br>n,abdomen (11)
train	12	breast cancer	300	268.0	m,lung (300)
train	16	lung cancer	75	254.0	m,lung (75)
train	22	colon cancer	46	471.6	m,liver (35)<br>m,lung (9)<br>m,pancreas (2)
train	23	colon cancer	31	148.9	m,suprarenal (3)<br>n,abdomen (2)<br>n,aortocaval (1)<br>n,iliac (2)<br>n,inguinal (2)<br>n,mesenteric (4)<br>n,pelvis (3)<br>n,retroperitoneal (14)
train	24	gastric cancer	17	238.8	m,liver (17)
train	25	gallbladder cancer	14	546.7	m,abdominal wall (4)<br>m,liver (3)<br>n,abdomen (1)<br>n,hepatic hilum (3)<br>n,periaortic (1)<br>n,retroperitoneal (2)
train	31	lung cancer	10	28.0	t,lung (4)<br>m,lung (6)
train	37	lung cancer	7	11.4	m,lung (4)<br>m,suprarenal (2)

					n,abdomen (1)
train	44	ovarian cancer	7	83.1	n,mesenteric (6)<br>n,retroperitoneal (1)
test	3	gallbladder cancer	9	424.5	n,abdomen (9)
test	10	melanoma	3	1.0	n,axillary (3)
test	13	gastric cancer	6	716.6	m,liver (6)
test	15	bladder cancer	9	26.7	n,pelvis (9)
test	18	colon cancer	7	82.8	n,abdomen (7)
test	28	colon cancer	584	1922.8	m,liver (82)<br>m,lung (488)<br>m,rib (1)<br>m,spleen (3)<br>n,abdomen (8)<br>n,pelvis (2)
test	30	rectal cancer	39	36.8	m,lung (39)
test	35	gastric cancer	6	288.8	m,abdominal wall (2)<br>m,ovary (4)

Supplementary Table 1. Annotated lesions for each patient.
